# Tunable electronic structures, Rashba splitting, and optical and photocatalytic responses of MSSe-PtO_2_ (M = Mo, W) van der Waals heterostructures

**DOI:** 10.1039/d3na00347g

**Published:** 2023-08-24

**Authors:** Sadia H. Gul, Tahani A. Alrebdi, M. Idrees, B. Amin

**Affiliations:** a Department of Physics, Abbottabad University of Science & Technology Abbottabad 22010 Pakistan binukhn@gmail.com +92-333-943-665 +92-333-943-665; b Department of Physics, College of Science, Princess Nourah Bint Abdulrahman University PO Box 84428 Riyadh 11671 Saudi Arabia; c School of Physics and Electronic Engineering, Jiangsu University Zhenjiang 212013 Jiangsu China

## Abstract

Binding energies, AIMD simulation and phonon spectra confirm both the thermal and dynamical stabilities of model-I and model-II of MSSe-PtO_2_ (M = Mo, W) vdWHs. An indirect type-II band alignment in both the models of MSSe-PtO_2_ vdWHs and a larger Rashba spin splitting in model-II than in model-I provide a platform for experimental design of MSSe-PtO_2_ vdWHs for optoelectronics and spintronic device applications. Transfer of electrons from the MSSe layer to the PtO_2_ layer at the interface of MSSe-PtO_2_ vdWHs makes MSSe (PtO_2_) p(n)-type. Large absorption in the visible region of MoSSe-PtO_2_ vdWHs, while blue shifts in WSSe-PtO_2_ vdWHs are observed. In the case of model-II of MSSe-PtO_2_ vdWHs, a further blue shift is observed. Furthermore, the photocatalytic response shows that MSSe-PtO_2_ vdWHs cross the standard water redox potentials confirming their capability to split water into H^+^/H_2_ and O_2_/H_2_O.

## Abstract 2

Photocatalytic response shows that bandedges of these vdWHs cross the standard water redox potentials confirming their capability to split water.

## Graphical abstract

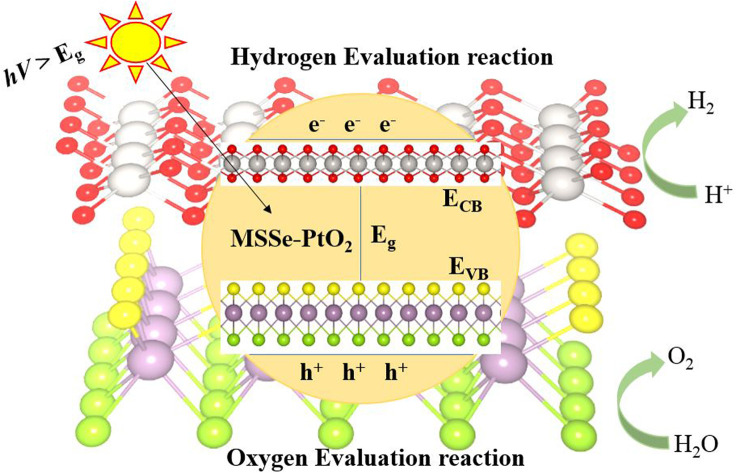

## 1. Introduction

Although the first 2D Janus material is graphone prepared using graphene through hydrogenation *via* DFT,^1,2^ Lu (Zhang) *et al.*^3,4^ have selenized (sulfurized) MoS_2_ (MoSe_2_) through the chemical vapor deposition (CVD) technique and named the resultant product MoSSe with MXY (M = Mo, W; (X ≠ Y) = S, Se) as the general formula. The space group of MoS_2_ is *D*_3*h*_ which also changed to *C*_3*v*_ for MXY monolayers with broken symmetry.^5,6^ Structural stability for MXY (M = Mo, W; (X ≠ Y) = S, Se, Te) *via* molecular dynamics and phonon dispersion spectra has been reported in ref. 7. SOC-induced Rashba spin splitting in the electronic band dispersion in specific monolayers of the MXY family shows potential for piezoelectricity and future spintronic device applications.^8–10^ Employing many-body Green's function perturbation theory, substantial excitonic effects are exhibited in the MoSSe monolayer used in optoelectronic devices.^11^ Recently, Paul *et al.*^12^ studied Janus PtXY (X, Y = S, Se, or Te) and observed that Rashba splitting is around the M point. They also investigated whether the band gaps and the strength of the Rashba effect could be further tuned through biaxial strain.

Heterostructures, fabricated by combining different 2D materials, are recognized as a helpful technique for tuning the electronic behavior of corresponding materials, for photovoltaic and nanoscale electronic devices.^13–16^ Vertically stacked layers are referred to as vdWHs as a result of the weak van der Waals (vdW) interactions with type-II band configuration,^17,18^ crucial for energy harvesting and photodetection.^19^ In type-II heterostructures, valence and conduction bands of one layer (layer A) are higher (in energy) than those of the other layer (layer B). The photo-generated electrons (holes) from the conduction (valence) band of layer A (B) migrate to layer B (A) which has low reduction (oxidation) potential, which results in spatial separation of electron–hole pairs, enhancing the performance of semiconductors in photocatalysis.^20–22^ VdWHs of TMDCs,^23^ MoS_2_–graphene,^24^ SiC-TMDCs,^25^ GeC-TMDCs,^26^ MoXY-WXY ((X ≠ Y) S, Se, Te),^27^ g-GaN-MSSe,^28^ BY-MX_2_ (Y = As, P),^29^ Janus Ga_2_SeTe/In_2_SSe,^30^ Janus-type platinum dichalcogenide heterostructures,^31^ PtS_2_/SnS_2_ (ref. 32), PtS_2_/arsenene^33^ and Janus-In_2_STe/InSe heterostructures^34^ have shown type-II band alignment with improved photocatalytic performance for water splitting. Li (Haleem) *et al.*^35,36^ presented a substantial Rashba spin splitting with a type-II band structure in MoSSe-WSSe (GeC-MSSe (M = Mo, W)) vdWHs, crucial for spintronic applications.

Another 2D material, PtO_2_, produced by exfoliation from bulk α-PtO_2_, survives at high temperature with better thermo-mechanical stability and superior optical absorption and carrier mobility.^37,38^ The PtO_2_ monolayer has been shown to be very closely matched with TMDCs to form vdWHs.^38^ It has been shown that PtO_2_–MoS_2_ (ZnO–PtO_2_) vdWHs with indirect (direct type-II) band alignment can be used in photocatalysis (photodetector) applications.^38,39^ Experimentally synthesized Ni(OH)_2_–PtO_2_ nanostructured arrays indicate enhanced hydrogen evolution reaction.^40^

Indeed, the small lattice mismatch, identical symmetry and high energetic feasibility of MSSe (M = Mo, W) and PtO_2_ led to the design of MSSe-PtO_2_ vdWHs. Two models of MSSe-PtO_2_ (M = Mo, W) vdWHs with four possible stacking configurations based on two different chalcogen atoms are fabricated. Both models of MSSe-PtO_2_ vdWHs with specific configuration are energetically, dynamically and thermally stable at 300 K. Furthermore, a detailed study is conducted to explore the electronic structure, Rashba spin splitting, work function, and optical and photocatalytic properties of the most stable configurations. Interestingly, we observed type-II band alignment in both models with considerable Rashba spin parameters and good photocatalytic response. These findings raised potential applications of MSSe-PtO_2_ vdWHs in nanoscale electronics, photovoltaics and photocatalysis.

## 2. Computational details

DFT with Grimme^41^ correction, a cut-off energy of 500 eV, a convergence criteria of 10^−3^ eV Å^−1^ (10^−4^ eV) for forces (energy), a *k*-mesh of 6 × 6 × 1 (12 × 12 × 1) and the PBE^42^ functional in the VASP^43,44^ are used for geometric relaxation (electronic structure calculations). Starting with converged PBE wave functions, the HSE06 functional^45^ without refining the *k*-mesh is also used for electronic band structure calculations. A 20 Å vacuum layer (to prevent the artifacts of the periodic boundaries along the *z*-axis) and the effect of SOC are also considered.^46^ Furthermore, using HSE06 wave functions, the Bethe–Salpeter equation was solved in the GW_0_ approach to study the imaginary part of the dielectric function.^47^

AIMD^48^ were performed using the Nose thermostat algorithm (with 300 K and a 1 fs time interval) to investigate the thermal stabilities of the above mentioned vdWHs. Dynamical stability of these systems was investigated by using density functional perturbation theory in the phonopy code, in which the harmonic interatomic force constant is used as the input^49,50^

## 3. Results and discussion

In agreement with ref. 51 and 52, optimized lattice constants (MoSSe ∼ 3.25 Å, WSSe ∼ 3.26 Å and PtO_2_ ∼ 3.17 Å) and bond lengths (M–S ∼ 2.411 Å, M–Se ∼ 2.39 Å and Pt–O ∼ 2.31 Å) show the reliability of our computational approach. MSSe and PtO_2_ have the same hexagonal lattice symmetry with a small and experimentally achievable lattice mismatch (MoSSe-PtO_2_ ∼ 2.46% and WSSe-PtO_2_ ∼ 2.27%), showing the possibility for experimental fabrication of vdWHs based on MSSe and PtO_2_ monolayers, *i.e.*, MSSe-PtO_2_ (M = Mo, W).^53^

**Fig. 1:**
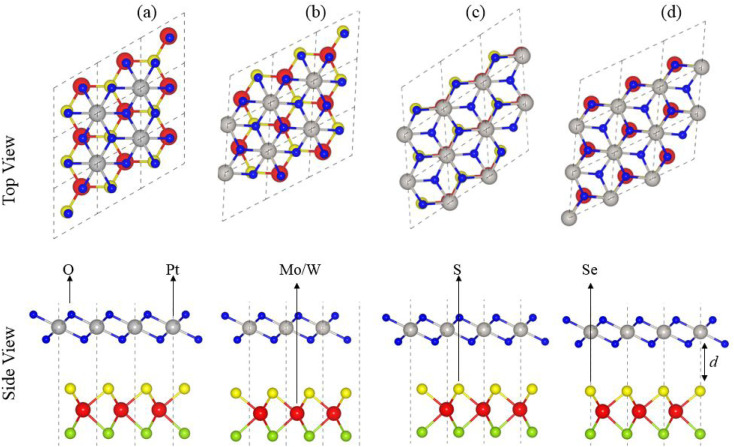
Model-I; stacking configuration of MSSe-PtO_2_ vdWHs.

As the interfacial properties are very sensitive to the layer configurations and connected atoms at the interface of vdWHs, in the case of the MSSe monolayer, two chalcogen atoms (S and Se) that terminate the surface are available for making vdWHs with the PtO_2_ monolayer. Therefore, we fabricated two different models of MSSe-PtO_2_ (M = Mo, W) vdWHs based on alternative chalcogen atoms. Each model has four possible stacking configurations, see model-I in Fig. 1. In model-I (a) stacking, the O atom of PtO_2_ is placed on top of the M atom of MSSe, while the S (Se) atom of MSSe and Pt atom of PtO_2_ are on the hexagonal site. In (b) stacking, the O atom of PtO_2_ is placed on top of the S (Se) atom of MSSe, while the M atom of MSSe and Pt atom of PtO_2_ are on the hexagonal site (just opposite of (a) stacking). In (c) stacking, the Pt atom of PtO_2_ is placed on top of the M atom of MSSe and one O atom of PtO_2_ is placed on top of the S (Se) atom of MSSe, while the other O is on the hexagonal site. In (d) stacking, the Pt atom of PtO_2_ is placed on top of the S (Se) atom of MSSe and one O atom of PtO_2_ is placed on top of the M atom of MSSe, while the other O atom is on the hexagonal site (just opposite of stacking (c)), see Fig. 1. Similar stacking configurations are also made for model-II with the alternative positions of S and Se atoms of MSSe layers.

Interlayer distance (d), binding energies (*E*_b_), optimized lattice constant (a) and bond length for each stacking configuration in both model-I and model-II of MSSe-PtO_2_ vdWHs in Table 1 are in the range of those of other vdWHs in ref. 27 and 28. Favorable stacking configuration ((a) stacking) in both models of MSSe-PtO_2_ vdWHs has the most negative binding energy and small interlayer distance, which shows higher energetic stability and strong physical and vdW interaction between MSSe and PtO_2_ layers. Slightly small vertical distances and high energetic feasibilities in model-II are due to the larger covalent radius of Se than the S atom at the interface which results in more attractive energy by making these stacking configurations, see Table 1. A small lattice mismatch induces minor strain in the corresponding monolayers (compressed Pt–O, while stretched M–S and M–Se bond lengths) of MSSe-PtO_2_ vdWHs, in agreement with ref. 26.

**Table 1:** Binding energies (*E*_b_ in eV), interlayer spacing (*d*_spacing_ in Å) for possible stacking configuration, lattice constant (*a* in Å), bond length (Pt–O, M–S, and M–Se in Å), bandgap (*E*_g_ in eV), Rashba parameter (*α*_R_ in eV), work function (*ϕ* in eV), potential drop (Δ*V* in eV), and effective mass (
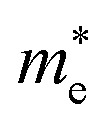
 and 
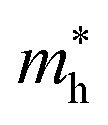
) for MSSe-PtO_2_ vdWHs

Heter	Model-I		Model-II	
	MoSSe-PtO_2_	WSSe-PtO_2_	MoSSe-PtO_2_	WSSe-PtO_2_
*E*_b_ (a)	−1.87	−1.54	−2.08	−1.70
*d*_spacing_	3.29	3.30	3.29	3.29
*E*_b_ (b)	−1.52	−1.20	−2.01	−1.59
*d*_spacing_	3.39	3.38	3.34	3.35
*E*_b_ (c)	−1.59	−1.32	−1.82	−1.47
*d*_spacing_	3.38	3.35	3.33	3.33
*E*_b_ (d)	−1.73	−1.29	−1.89	−1.61
*d*_spacing_	3.31	3.36	3.31	3.32
*a*	3.21	3.215	3.21	3.215
Pt–O	2.064	2.064	2.064	2.064
M–S	2.411	2.415	2.411	2.415
M–Se	2.527	2.532	2.527	2.532
*E*_g-(PBE)_	0.62	0.494	0.287	0.204
*E*_g-(HSE06)_	1.31	0.97	0.61	0.51
*α*_R-(PBE)_	0.61	0.75	0.69	0.83
*α*_R-(HSE06)_	0.68	0.89	0.75	0.97
*ϕ*	2.01	2.05	2.26	2.31
Δ*V*	5.862	5.72	6.506	6.387
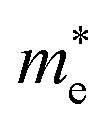	0.593	0.514	0.483	0.419
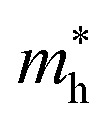	0.758	0.980	1.292	1.863

**Fig. 2:**
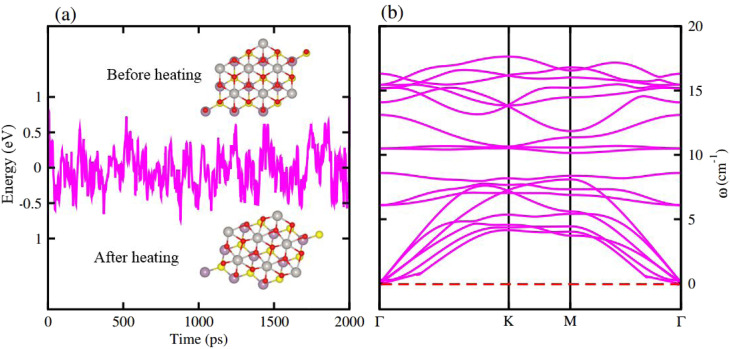
AIMD simulation (a) and phonon spectrum (b) of MoSSe-PtO_2_ vdWHs in model-I.

AIMD simulations for the most stable stacking configuration ((a) stacking) of MoSSe-PtO_2_ vdWHs in model-I, in Fig. 2(a), show a small variation in the total energy, while no distortion was found in the structure after 6 ps, hence confirming the thermal stability at 300 K. The phonon band structure of the same vdWH for model-I, in Fig. 2(b), is free from imaginary frequency, hence confirming the dynamic stability of MSSe-PtO_2_ (M = Mo, W) vdWHs. Similar trends were also found for other vdWHs in both model-I and model-II.

**Fig. 3:**
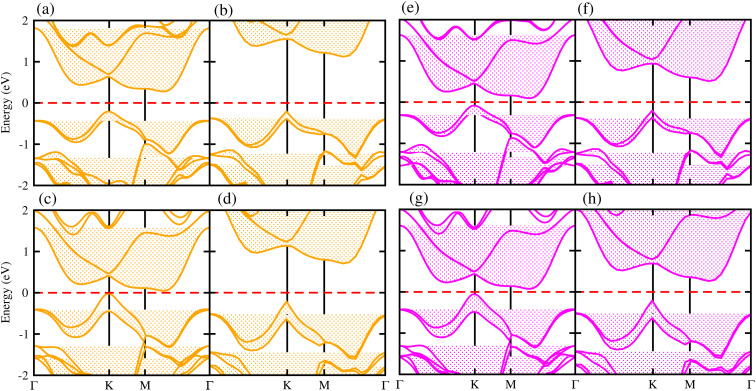
Electronic band structures of MoSSe-PtO_2_ (a(b) and e(f)) and WSSe-PtO_2_ vdWHs (c(d) and g(h)), using the PBE (HSE06) method. The left(right) column is for model-I (-II).

**Fig. 4:**
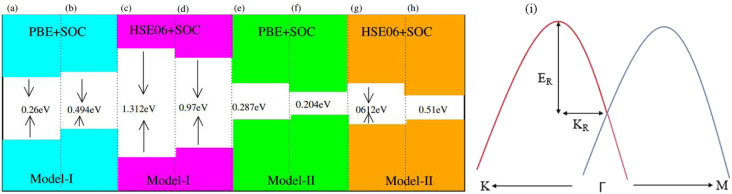
A sample texture for (a–h) bandgap and (i) Rashba spin splitting.

Generally, in DFT, the choice of the functional (conventional or hybrid) affects the band structure and bandgap values. Therefore, we have used both PBE and HSE06 functionals to calculate the electronic band structure of MSSe-PtO_2_ vdWHs, see Fig. 3. In both models, the VBM (CBM) is pinned at the K (M-*Γ*)-point of the first BZ and hence reveals the indirect bandgap nature of MSSe-PtO_2_ vdWHs. The bandgap values calculated by the HSE06 method are larger than those obtained by the calculations at the PBE level, which are further decreased by including the SOC effect due to the VBM/CBM spin splitting and mirror symmetry breaking in MSSe monolayers, see Table 1 and Fig. 4(a)–(h). The bandgap values also decrease from Mo to W and also from model-I to model-II which may be due to the different chalcogen atoms attached to PtO_2_ layers in MSSe-PtO_2_ vdWHs. Replacing one of the similar chalcogen atoms in the MX_2_ monolayer by a different one, with vertical stacking in vdWHs, breaks the inversion symmetry, due to which an electric field is produced and generates Rashba spin splitting at the *Γ*-point near the Fermi level (see Fig. 3 and schematic representation in Fig. 4(i));^54^ calculated using *α*_R_ = 2*E*_R_/*K*_R_. *E*_R_ (*K*_R_) is the Rashba energy (moment offset at the *Γ*-point, known as the Rashba wave vector). *α*_R_ values calculated for MSSe-PtO_2_ vdWHs in model-I (II) are presented in Table 1. It is important to address that *α*_R_ for MSSe-PtO_2_ vdWHs is smaller than that of the corresponding isolated monolayer,^26^ which may be due to the intrinsic electric field.^55^ Furthermore, the *α*_R_ values increase from model-I to -II and also from MoSSe-PtO_2_ to WSSe-PtO_2_ vdWHs which is due to the selective atom of chalcogen atom and heavier W atom than the Mo atom. Hence, MSSe-PtO_2_ vdWHs with considerable *α*_R_ values set a platform for practical application in spintronic devices.

**Fig. 5:**
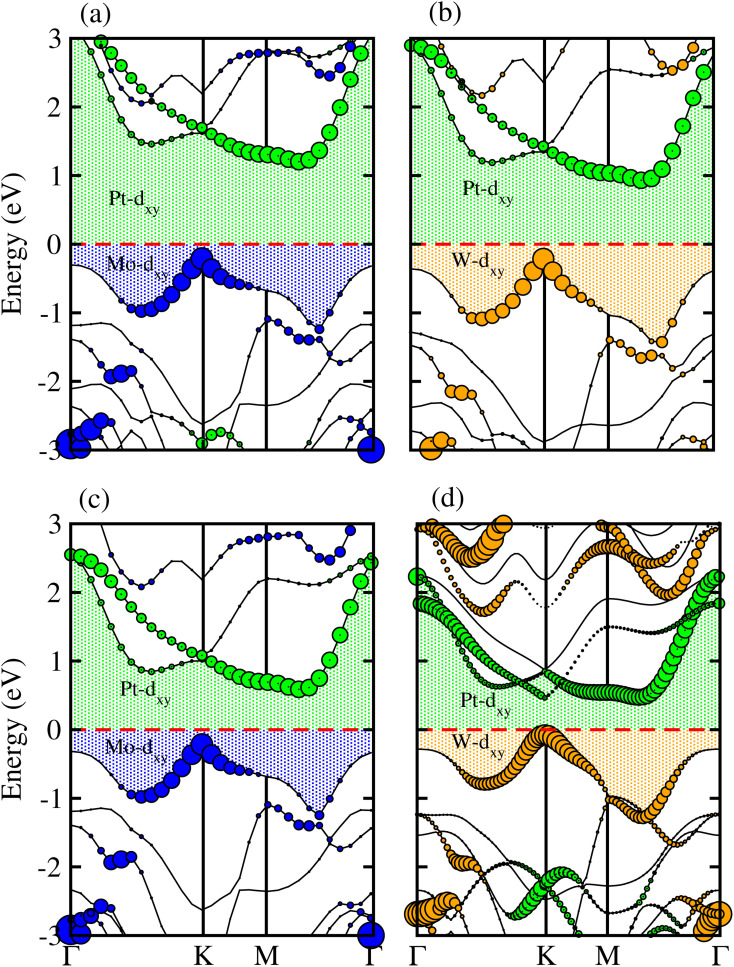
Weighted band structure of MSSe-PtO_2_ vdWHs in model-I (a(b)) and in model-II (c(d)).

Partial band structures of MSSe-PtO_2_ (M = Mo, W) vdWHs, depicted in Fig. 5, show that the M-d_*xy*_ (Pt-d_*xy*_) state of the MSSe (PtO_2_) layer mainly contributes to the VBM (CBM) of MSSe-PtO_2_ vdWHs. Localization of the VBM and CBM from the isolated MSSe and PtO_2_ layers confirms type-II (staggered) band alignment in MSSe-PtO_2_ vdWHs, responsible for charge carrier separation.^35,36^ Hence in the case of MSSe-PtO_2_ vdWHs, photogenerated carriers move from different layers (electrons (holes) move from the MSSe (PtO_2_) layer to the PtO_2_ (MSSe) layer), which may decrease their recombination rate and hence play an important role in photocatalysis and solar cell application.

**Fig. 6:**
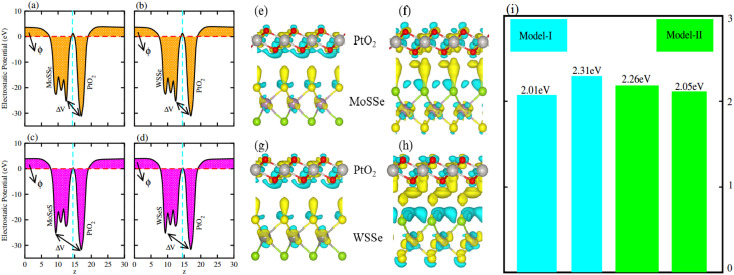
Electrostatic potential in model-I (a(b)) and in model-II (c(d)) and the charge density difference in model-I (e(g)) and in model-II (f(h)) of MSSe-PtO_2_ vdWHs, where (i) represents the calculated work function of the corresponding materials.

Average electrostatic potential (Fig. 6(a)–(d)) and the charge density difference (CDD) (Fig. 6(e)–(h)) are also calculated to understand both the qualitative and quantitative behaviour of the transfer of charge among the layers of MSSe-PtO_2_ (M = Mo, W) vdWHs. One can see that the PtO_2_ layer has deeper potential than the MSSe layer, indicating that electrons are moving from the MSSe layer to the PtO_2_ layer at the interface of MSSe-PtO_2_ vdWHs. The CDD in Fig. 6(e)–(h) confirms the charge transfer from the MSSe layer to PtO_2_; hence the MSSe (PtO_2_) monolayer becomes p(n)-doped after stacking. The deeper potential of S than Se is due to the electronegativity difference. Potential drops between MSSe and PtO_2_ layers, presented in Table 1 for model-I and model-II, differentiate the excitonic behavior of free-standing monolayers from the corresponding vdWHs, which helps promote photogenerated carrier (electrons and holes) separation. Quantitative charge transfer is also investigated by Bader charge analysis, which shows that about 0.086*e* (0.097*e*) and 0.075*e* (0.91*e*) are transferred from the MoSSe and WSSe layer to the PtO_2_ layer in model-I (-II) of MSSe-PtO_2_ vdWHs. This small transfer of charges from the MSSe layer to the PtO_2_ layer confirms weak vdW interactions between the layers of heterostructures. The work function (*ϕ*) is also calculated by using *ϕ* = *E*_vac_ − *E*_F_, where *E*_vac_ is the energy difference of the vacuum potential (derived from the electrostatic potential (see Fig. 6(i)) in the direction normal to the surface with large vacuum separation) to Fermi energy *E*_F_ (given from the ground state DFT calculations). The calculated values for *ϕ* in Table 1 are efficient for formation of the interface and charge transfer. One can observe that the values of *ϕ* increase from model-I to model-II and also from MoSSe-PtO_2_ to WSSe-PtO_2_ vdWHs, which may be due to the selective atom of chalcogen atom and heavier W atom than the Mo atom. Fabrication of MSSe-PtO_2_ (M = Mo, W) vdWHs modulates the band structure of the corresponding monolayers, hence the effective masses. Therefore, we have calculated the effective mass of MSSe-PtO_2_ (M = Mo, W) vdWHs by using parabolic fitting to the band edge, given in ref. 56; 
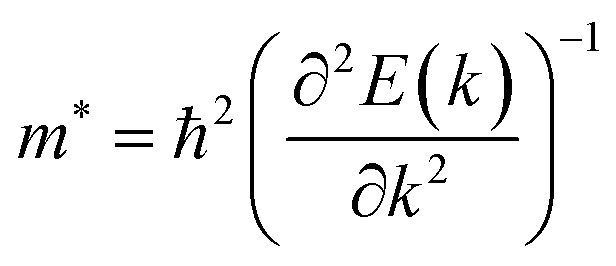
. The calculated effective mass of electrons (holes) is 
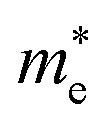
 (
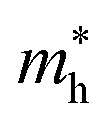
), given in Table 1. One can see that the 
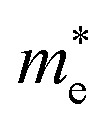
 values are smaller than the corresponding 
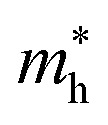
 values, while smaller values for 
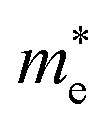
 are further observed in WSSe-PtO_2_ (M-II) and for 
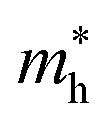
 in MoSSe-PtO_2_ (M-I). High (smaller) carrier mobility (effective mass) is preferable for electronic and optoelectronic devices.^57^ From Table 1 it’s clear that WSSe-PtO_2_ (M-I) has miniaturized effective masses, hence sponsors high charge carrier mobility, and therefore, shows good response towards high performance device applications.^57^

**Fig. 7:**
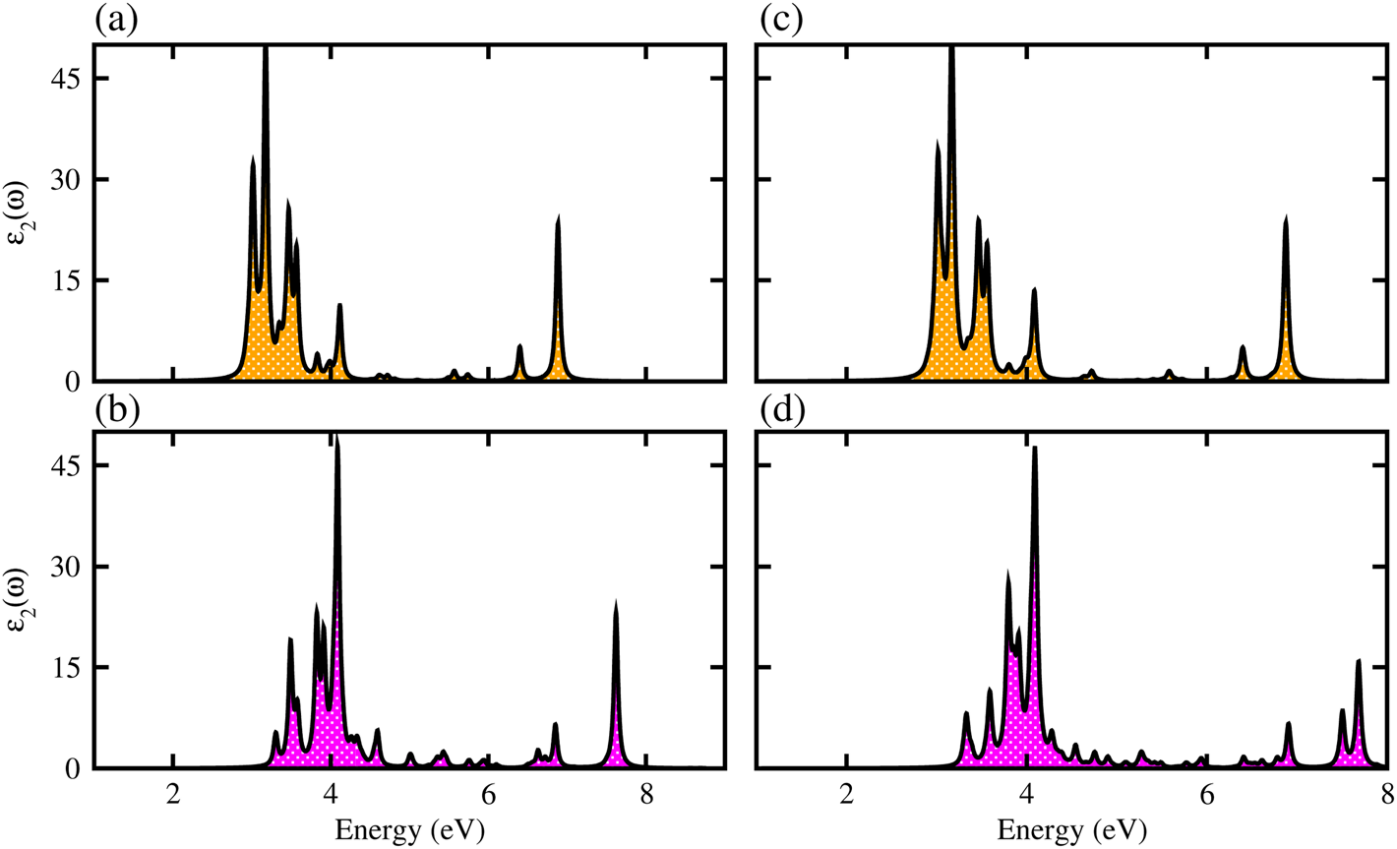
*ε*_2_ (*ω*) of MoSSe-PtO_2_ (WSSe-PtO_2_) vdWHs (a(b)) in model-I and (c(d)) in model-II.

We have further explored the optical response of MSSe-PtO_2_ vdWHs in terms of *ε*_2_ (*ω*), directly associated with the band structure and the bandgap values,^58,59^ shown in Fig. 7. The optical absorption range for MoSSe and WSSe monolayers is about 2 to 5.0 eV,^27^ while for Mo(W)SSe-PtO_2_ vdWHs in model-I and model-II it is from 2.5 (3.0) to 7.0 (8.0) eV. Broader absorption in the visible region of MoSSe-PtO_2_ than WSSe-PtO_2_ vdWHs is due to the smaller bandgap of the former, while a blue shift is observed in the latter. A further blue shift in model-II than in model-I of MSSe-PtO_2_ vdWHs may be due to the selective atom of chalcogen atom at the interface. Although there is a blue shift in the absorption spectrum of MSSe-PtO_2_ vdWHs from model-I to -II, the qualitative behaviour of the peak is similar for both models. High optical absorption requires a wide range peak, which may occupy a large number of states near the Fermi level. Broadened optical absorption of vdWHs is due to high carrier density (compared to parent monolayers).^60^ Obviously, the fabrication of vdWHs is an effective way to understand the modulation of absorption performance of 2D layered materials. We predict that MoSSe-PtO_2_ vdWHs for model-I (-II) have good optical absorption in visible regions, which makes them suitable for practical applications in nanoelectronics and optoelectronic devices.^61^

**Fig. 8:**
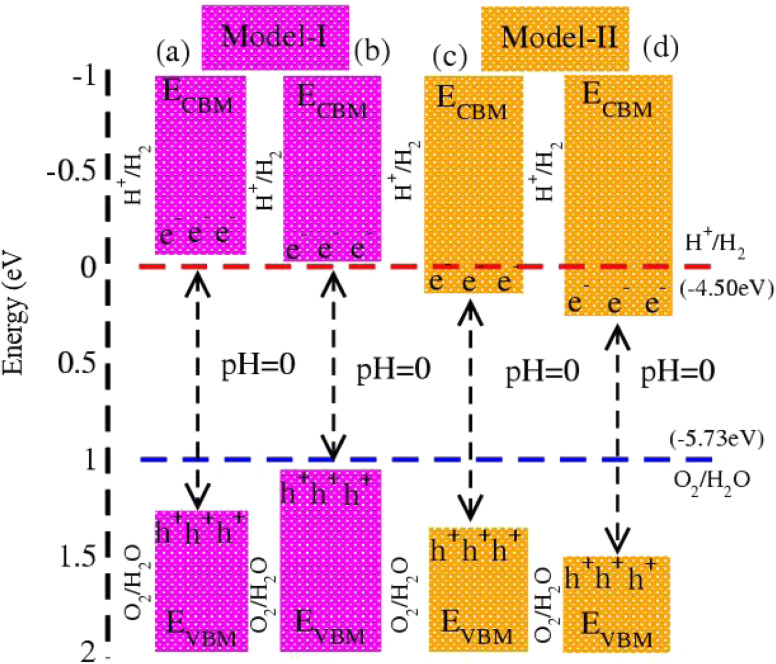
Photocatalytic water splitting of MSSe-PtO_2_ vdWHs, where blue and red dotted lines represent reduction and oxidation potentials at pH = 0.

In photocatalytic activity, illumination of solar light on semiconductors separates charge carriers in conduction and valence bands. Using Mulliken electronegativity (*χ*)^62^ and standard electrode potential on the hydrogen scale (*E*_elec_ = 4.5 eV), energies of valence (*E*_VBM_) and conduction (*E*_VBM_) band edge potentials at pH = 0 are *E*_VBM_ = *χ* − *E*_elec_ − 0.5 *E*_g_ and *E*_CBM_ = *E*_VBM_ − *E*_g_. Standard water redox potentials are −4.50 (−5.73) eV for reduction (oxidation) *i.e.*, H^+^/H_2_ (H_2_O/O_2_),^23,63^ which show that the size of the bandgap and energy of the band edge potentials are the basic criteria to facilitate water splitting reactions. Both *E*_VBM_ and *E*_CBM_ of MSSe-PtO_2_ (M = Mo, W) vdWHs in model-I and model-II are presented in Fig. 8. In the case of model-I of MSSe-PtO_2_ vdWHs, both *E*_VBM_ and *E*_CBM_ cross the standard redox and oxidation potential at pH = 0, and hence show the capacity for full water splitting. In model-II of MSSe-PtO_2_ vdWHs, *E*_VBM_ crosses standard redox potential, hence having the ability to reduce water at H^+^/H_2_ but failing for oxidation (O_2_/H_2_O). It is clear from the above discussion and Fig. 8 that photocatalytic water splitting activity is very sensitive to the order of chalcogen atoms attached at the interface of vdWHs. Similar photocatalytic water splitting is also demonstrated in ref. 29.

## 4. Conclusion

In summary, using first principles calculations, electronic structures, Rashba splitting, and optical and photocatalytic properties of MSSe-PtO_2_ (M = Mo, W) vdWHs are investigated. Binding energies, AIMD simulation and phonon spectra confirm the thermal and dynamical stabilities of MSSe-PtO_2_ vdWHs in model-I and model-II. Electronic band structures show that MSSe-PtO_2_ vdWHs are indirect type-II semiconductors. Interestingly Rashba spin splitting observed in both model-I and model-II of MSSe-PtO_2_ vdWHs, with larger Rashba parameters in model-I than in model-II, provides a platform for experimental design of MSSe-PtO_2_ vdWHs for optoelectronics and spintronic device applications. Transfer of electrons from the MSSe layer to the PtO_2_ layer at the interface of MSSe-PtO_2_ vdWHs makes the MSSe (PtO_2_) monolayer p(n)-doped after stacking. Broad absorption occurs in the visible region of MoSSe-PtO_2_ vdWHs and a blue shift is observed in WSSe-PtO_2_ vdWHs. A further blue shift is also observed in model-II than in model-I of MSSe-PtO_2_ vdWHs. MSSe-PtO_2_ vdWHs in model-I are also found to be exciting materials for water splitting and suggested for low cost hydrogen production.

## Conflicts of interest

There are no conflicts to declare.

## Supplementary Material
